# Protocol for a randomised controlled trial of a decision aid for the management of pain in labour and childbirth [ISRCTN52287533]

**DOI:** 10.1186/1471-2393-4-24

**Published:** 2004-12-09

**Authors:** Christine L Roberts, Camille H Raynes-Greenow, Natasha Nassar, Lyndal Trevena, Kirsten McCaffery

**Affiliations:** 1 Centre for Perinatal Health Services Research, QEII Building DO2, University of Sydney, NSW 2006, Australia; 2 School of Public Health, Edward Ford Building A27, University of Sydney NSW 2006, Australia

## Abstract

**Background:**

Women report fear of pain in childbirth and often lack complete information on analgesic options prior to labour. Preferences for pain relief should be discussed before labour begins. A woman's antepartum decision to use pain relief is likely influenced by her cultural background, friends, family, the media, literature and her antenatal caregivers. Pregnant women report that information about analgesia was most commonly derived from hearsay and least commonly from health professionals. Decision aids are emerging as a promising tool to assist practitioners and their patients in evidence-based decision making.

Decision aids are designed to assist patients and their doctors in making informed decisions using information that is unbiased and based on high quality research evidence. Decision aids are non-directive in the sense that they do not aim to steer the user towards any one option, but rather to support decision making which is informed and consistent with personal values.

**Methods/design:**

We aim to evaluate the effectiveness of a Pain Relief for Labour decision aid, with and without an audio-component, compared to a pamphlet in a three-arm randomised controlled trial. Approximately 600 women expecting their first baby and planning a vaginal birth will be recruited for the trial.

The primary outcomes of the study are decisional conflict (uncertainty about a course of action), knowledge, anxiety and satisfaction with decision-making and will be assessed using self-administered questionnaires. The decision aid is not intended to influence the type of analgesia used during labour, however we will monitor health service utilisation rates and maternal and perinatal outcomes. This study is funded by a competitive peer-reviewed grant from the Australian National Health and Medical Research Council (No. 253635).

**Discussion:**

The Pain Relief for Labour decision aid was developed using the Ottawa Decision Support Framework and systematic reviews of the evidence about the benefits and risks of the non-pharmacological and pharmacological methods of pain relief for labour. It comprises a workbook and worksheet and has been developed in two forms – with and without an audio-component (compact disc). The format allows women to take the decision aid home and discuss it with their partner.

## Background

### Patient participation in clinical decision making

Making evidence-based decisions in clinical practice is not always straightforward: patients and their healthcare providers may need to weigh up the evidence between several comparable options, the evidence for some treatments may be inconclusive, and the information needs to be tailored to each patient's clinical context and personal preferences [1,2]. Good medical decision making should take into account the best available evidence, along with patients' preferences and values [3]. However, finding effective and efficient mechanisms for doing this in the clinical setting is a challenge.

To assist patients and their doctors in making informed decisions, information must be unbiased and based on current, high quality, quantitative research evidence. However, patient information materials are often outdated, inaccurate, omit relevant data, fail to give a balanced view and ignore uncertainties and scientific controversies [4,5]. It is increasingly evident that the provision of patient and provider information alone, even if evidence-based, is not sufficient to influence health outcomes and behaviour [6]. It is only when mechanisms are provided that tailor this information to the individual patient that health outcomes, related to treatment decisions, are positively effected [7]. With this in mind, decision aids are emerging as a promising tool to assist practitioners and their patients in evidence-based decision making [1].

### Decision Aids

Decision aids are "interventions designed to help people make specific and deliberative choices among options by providing (at minimum) information on the options and outcomes relevant to the person's health status" [1]. Additional strategies may include providing: information on the condition; the probabilities of outcomes tailored to a person's health risk factors; an explicit values clarification exercise; examples of others' decisions; and guidance in the steps of decision making [1]. Decision aids are non-directive in the sense that they do not aim to steer the user towards any one option, but rather to support decision making which is informed, consistent with personal values and acted upon [1]. Decision aids have been found to improve patient knowledge and create more realistic expectations, to reduce decisional conflict (uncertainty about the course of action) and to stimulate patients to be more active in decision making without increasing anxiety [1].

Internationally decision aids have been evaluated in a variety of health and clinical settings. Although their use in pregnancy and birth has only just begun to be explored, this is an area in which consumers are known to want to participate actively in decision making [8]. A survey of 790 Australian women reported a tenfold increase in dissatisfaction among women who did not have an active say in decisions about pregnancy care [8]. Similarly in the UK, women rated the explanation of procedures, including the risks, before they are carried out and involvement in decision making as most important to satisfaction with care [9]. Significantly, neither obstetricians nor midwives appreciated the importance to women of "being told the major risks for each procedure" [9]. Our own survey of pregnant women attending an antenatal clinic found that overwhelmingly women wanted to be involved in decisions regarding their pregnancy care, and this was regardless of age, parity, education or delivery preferences [10].

### Labour pain

The pain of labour is a central part of women's experience of childbirth and is a constant feature of antenatal discussion groups [11]. Most women giving birth use some methods of pain relief (pharmacologic and/or non-pharmacologic) during labour. In Australia 92% of primiparas and 71% of multiparas use some analgesic agents for labour analgesia [12]. Significantly, there have been more clinical trials of pharmacological pain relief during labour and childbirth than of any other intervention in the perinatal field [13].

However satisfaction with childbirth is not necessarily contingent upon the absence of pain [14]. Many women are willing to experience pain in childbirth but do not want pain to overwhelm them. The Royal College of Obstetrics and Gynaecology (RCOG) makes the following evidence-based recommendations [15]:

• Continuous caregiver support for a single individual should be available to women in labour

• Midwives must involve women in decisions about analgesia and recognise the value of promoting personal control

• Maternity services should ensure access to written and verbal information on pain relief and should support women in their choices for pain relief

• Maternity services should respect women's wishes to have some control over their pain relief

• Improved public information and data on pain and analgesia

In Australia over 250,000 women give birth annually and the increasing use of epidural analgesia means some 75,000 women have an epidural in labour each year [16]. Among primiparas in NSW, the epidural rate increased from 25% in 1990 to 42% in 2000, but was as high as 74% in hospitals with greater availability of epidurals [12]. Other pharmacologic methods of pain relief for primiparas include 36% opioids and 55% nitrous oxide [12].

### Pharmacologic methods of pain relief in labour and childbirth

Randomised controlled trials have shown epidural analgesia provides the most efficacious pain relief for labour, but the adverse consequences include prolonged labour, restricted mobility, use of oxytocin augmentation and an increased incidence of instrumental delivery [17,18]. Consequences of instrumental delivery at 6 months postpartum include perineal pain 54%, urinary incontinence 18%, bowel problems 19%, haemorrhoids 36% and sexual problems 39% [19]. Further, the complications of epidurals can include unsatisfactory analgesia, dural-puncture headache, hypotension, nausea/vomiting, fever, localised backache, shivering, pruritis and urinary retention [18].

Although not as effective as epidural, randomised trials show inhalational analgesia (e.g. 50% nitrous oxide in oxygen) and systemic opioid analgesics (e.g. pethidine) can provide modest benefit to some patients during labour or supplement an unsatisfactory epidural [13]. Both these methods can cause nausea, vomiting and dizziness, and additionally opioid side-effects may include orthostatic hypotension, delayed stomach emptying and respiratory depression in the baby [13].

### Non-pharmacologic methods of pain relief in labour and childbirth

A number of women prefer to avoid pharmacological analgesia if possible [20]. The wish to maintain personal control during labour and birth, the desire to participate fully in the experience, and concerns about untoward effects of medications during labour, are among the factors that influence their attitude [20]. Non-pharmacological methods of pain relief include maternal movement and position changes, superficial heat and cold, immersion in water*, massage, acupuncture/acupressure, transcutaneous electrical nerve stimulation (TENS)*, aromatherapy, attention focussing, hypnosis*, music/audioanalgesia* and continuous caregiver support*. Only a few of these methods (marked*) have been assessed in randomised trials [20-22]. Only continuous caregiver support resulted in reduced analgesia requirements (and length of labour and the incidence of operative delivery). Although the other interventions trialled did not reduce the use of pharmacologic analgesia, they were well liked by women and had few side effects.

### Decision making and pain in labour

Women report fear of pain in childbirth and often lack complete information on analgesic options prior to labour [11]. For example a Royal Australian and New Zealand College of Obstetrics and Gynaecology brochure on 'Epidural and Spinal Anaesthesia' reports the advantages of epidurals but does not mention any possible adverse outcomes or complications [23]. While written informed consent is required for epidural analgesia, it is not required for other analgesic options. Further, the consent for epidural (covering only the procedure and complications) is obtained by the anaesthetist at the time of the procedure – by which time most women are already distressed [24].

Dickerson stresses the importance of discussing preferences for pain relief before labour begins [13]. A woman's antepartum decision to use pain relief is likely influenced by her cultural background, friends, family, the media, literature and her antenatal caregivers [25]. A survey of Australian women found that antepartum information about analgesia was most commonly derived from hearsay and least commonly from health professionals [26]. Antenatally 82% of women wish to see how labour progresses and only want analgesia when pain becomes severe or intolerable [14]. Antenatal plans for analgesia are strongly associated with use: 96% of women who definitely planned to have an epidural, received one [25].

The management of pain in labour is a clinical decision that fulfils Eddy's criteria for a decision in which patients' values and preferences should be included [2]. The outcomes for analgesia options and, women's preferences for the relative value of benefits compared to risks are variable and could result in decisional conflict. For such a clinical decision, a decision aid would be expected to improve patient knowledge and create realistic expectations, to reduce decisional conflict and to stimulate patients to be more active in decision making without increasing anxiety [1]. Leap has suggested a 'working with pain' framework for managing labour and childbirth in a positive context [11]. This framework which aims to develop an understanding of 'normal pain' as part of the process of labour, rather than the absolute amelioration of pain, has been recommended by the Royal College of Obstetrics and Gynaecology.

### Development of a decision aid on the management of pain during labour

During 2003 and 2004, we developed an evidence-based decision aid about the management of pain in labour for women having their first baby. This followed a needs assessment that collected data on the attitudes, preferences and knowledge of nulliparous women who were making plans about pain relief for labour and childbirth. The needs assessment found that women's knowledge of pain relief options was limited and these women would benefit from a decision aid for labour analgesia.

In developing the decision aid we utilised the NHMRC guideline "How to prepare and present information for consumers of health services" [27] and the Ottawa framework established and rigorously tested by the Ottawa Health Decision Center [28]. The decision aid was developed to incorporate a workbook (with and without a complementary audio-component as a compact disc) and worksheet. The workbook highlights key points (similar to a slide presentation) and the audio component connects these points in a narrative format, providing more detail than the workbook. The worksheet is a one-page sheet to be completed by the woman to record her decision making steps, to list any questions she needs answered before deciding, and to encourage her to discuss he plans with her labour care providers. Most importantly, the decision aid is intended to be non-directive in that it does not aim to steer the user towards any one option or increase or decrease intervention rates but rather act as an adjunct to care

The decision aid was designed for women to use at home or in the clinical setting, and takes about 30 minutes to complete. After working through the decision aid, women should take the completed worksheet to their next antenatal appointment to discuss their preferences with their health care provider. The worksheet is also useful for the practitioner, who can see rapidly from it what evidence the patient has considered, what her values and preferences are and which way she is leaning in her preferences for analgesia during labour.

The decision aid was developed, pilot tested and revised with extensive consumer involvement, as outlined in the NHMRC guideline on preparing information for consumers [27]. The content of the decision aid was largely driven by consumers' questions and information needs as determined from the focus groups and from the process of drafting, pilot testing and re-drafting.

A number of draft decision aids (including workbook, audio transcript, and worksheet), were developed and each subjected to pilot testing and revision as we obtained feedback. The process of testing and revising started with the study project group. The next phase included a review by a group of national and international content experts, including decision aid experts, obstetricians, midwives, perinatal epidemiologists, parent educators and psychologists. Once we were convinced that the content was accurate the decision aid was pilot-tested amongst consumers. There were several rounds of consumer review and refinement.

Initially we aimed to compare the Decision Aid (workbook and audio-component) with usual care and counselling however preliminary work led us to alter our original study design. We could find no studies that compared Decision Aids with and without an audio-component. As the audio-component adds considerable complexity to the development and cost of the Decision Aid we decided to have two intervention arms: a Decision Aid with an audio-component and a Decision Aid without an audio-component. Further in pilot testing we found that women in the usual care arm were disappointed to not receive any information. Thus, to minimise refusals and losses to follow-up we decided to issue the women in the control group with a pamphlet called "Pain relief during childbirth – A guide for women" This pamphlet is published by the Royal Australian and New Zealand College of Obstetricians and Gynaecologists, is publicly available and includes information about methods of pain relief during labour [29]. These changes to the study protocol were approved by the institutional ethics committee prior to commencement of the trial.

## Methods/design

### 1. Specific Aim

To compare the relative effectiveness of the Pain Relief for Labour Decision Aid with a pamphlet on women's decisional conflict, knowledge, expectations, satisfaction with decision making and anxiety, and examine its impact on service utilisation and perinatal outcomes (as secondary outcomes).

### 2. Hypotheses

The primary study hypotheses are:

Use of the Pain Relief for Labour Decision Aid by women expecting their first baby:

1. Reduces decisional conflict (uncertainty about the course of action)

2. Increases knowledge of labour analgesia

3. Increases satisfaction with their decision making

4. Reduces anxiety.

The secondary hypotheses of the study are:

Use of the Pain Relief for Labour Decision Aid by women expecting their first baby will not influence:

1. The type of analgesia women use for labour

2. Maternal and infant outcomes.

### 3. Study design

We will conduct a randomised trial with the following study groups to assess the impact of the decision aid:

Group 1: The pamphlet, "Pain relief during childbirth – A guide for women" [29]

Group 2: Decision aid with an audio-component

Group 3: Decision aid without an audio-component

### 4. Setting

An Australian tertiary obstetric hospital with a full range of non-drug and anaesthetic options for pain relief in labour. Epidurals are available 24 hours a day from anaesthetic staff designated to labour ward. All forms of antenatal care (clinic, birth centre, private, shared care with a family physician) will be included in the study.

### 5. Participants/eligibility criteria

Primiparous women in late pregnancy (≥36 weeks gestation) who are expecting to have a vaginal birth of a single infant will be eligible for the study. Primiparous women were selected because previous pregnancies have a strong impact on decision making and analgesia use in labour [14,16]. Exclusions include women who will not have any choice about analgesia, for example planned caesarean section (eg breech, placenta praevia, HIV), planned epidural (eg symptomatic heart disease), contraindications to analgesia (e.g drug sensitivities, anticoagulants, thrombocytopaenia). The decision aid was produced in English and designed to be simple and accessible for women with low levels of literacy.

### 6. Procedures, recruitment, randomisation and collection of baseline data

**Figure 1:**
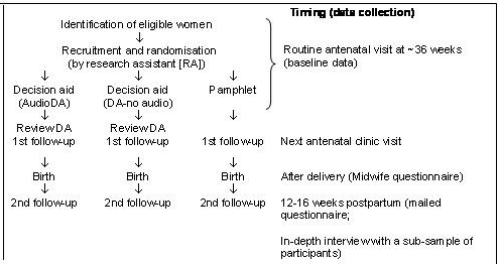
Schema of Pain Relief for Labour Decision Aid trial

The study procedure draws on the usual schedule of weekly antenatal visits in late pregnancy (Figure 1). We plan a pragmatic approach to assess the decision aid under the conditions most likely to be applied in practice. A research nurse will ask eligible women to participate, explain the trial and obtain informed consent, collect baseline data and randomly allocate women (using telephone randomisation) to one of the study groups. This is only a minor deviation from current practice. As women of child-bearing age are known to be very mobile, participants will be asked to provide alternate contact details (eg friend or relative) to enhance subsequent follow-up. Private obstetricians will be asked to offer participation in the study to their patients. Those interested will be requested to come to the antenatal clinic for recruitment and randomisation. The private obstetrician will provide standard care. Flyers and posters will be prepared to inform women of the study and will be distributed through family physicians and obstetricians as well as the clinics.

Brief baseline data will be collected to assess comparability of the study groups. The baseline assessment will include age, brief socio-demographic data, highest level of education achieved, anxiety as assessed by the state component of the short Spielberger anxiety scale [30], and information sources about labour analgesia.

### 7 Intervention

The aim of the decision aid is to assist preference elicitation, and not to influence the direction of the decision taken. Women in each study group will be given the opportunity to review the intervention they are allocated (decision aid or pamphlet) while in the antenatal clinic and/or to take home, which ever is most convenient. Many women will also want to discuss their preferences with their partner. At the next antenatal visit, women will be contacted by the research nurse to discuss the information materials and any questions they may have had.

### 8 Follow-up

#### i) First follow-up questionnaire

All participants will be given a follow-up questionnaire prior to their next antenatal consultation. (See Outcome Measure details below).

#### ii) Midwife questionnaire

After a study participant delivers, the midwife who provided the labour care will complete a brief questionnaire to assess the impact of the decision aid on the management of labour analgesia. Information will also be collected on caregiver support in labour, birthplace (delivery suite or birth centre), use of non-drug analgesic options and stage of labour at admission.

#### iii) Second follow-up questionnaire

At 12–16 weeks postpartum all participants will be mailed a second follow-up questionnaire. This will assess women's satisfaction with the decisions made and the decision-making processes. (See Outcome Measures below). Questionnaires will be mailed with reply paid envelopes, with up to two reminder telephone prompts to non-responders.

#### iv) Qualitative follow-up

We will conduct in-depth interviews to explore the impact of the decision aid on women's experiences in labour and childbirth. A sub-sample of 30 women will be purposively selected, to reflect heterogeneity of experience of labour. The interviews will provide an understanding of the complexities of analgesic preferences, management, expectations, satisfaction, and psychological health following delivery. This data will enable examination of unpredicted and subtle effects of the decision aid on psychosocial outcomes that may not be captured using quantitative methods. Interviews will be face-to-face and conducted in women's homes or at a clinic, according to participants' preferences. Interviews will be recorded and transcribed. Data will be analysed using thematic analysis.

### 9. Blinding and contamination

As with many obstetric interventions blinding is virtually impossible. The main outcomes of this study are self-reported and the women are clearly not blinded to their treatment allocation. However, we will institute a number of measures aimed at keeping antenatal staff blind to the treatment allocation and preventing contamination of the control group:

• Women will review the decision aid with the research nurse and complete the first questionnaire (primary outcome measures) prior to their next antenatal consultation

• Usual antenatal care providers will be blinded to the exact content and format of the decision aid

• Regular in-service (educational training) for the antenatal care providers to explain the trial protocol and to make clear the potential effect of unmasking or contamination.

• Monitoring decision aid distribution and keeping them locked up and only accessible by the research nurse

• Asking participants not to reveal their treatment allocation, or share their decision aid material with antenatal staff or other women. If participants do not want to keep their decision aid they will be asked to return it.

### 10 outcome measures

#### Primary outcomes

The primary outcomes of this study will be:

*Decisional conflict *(uncertainty about which preference to choose) will be assessed by the Decisional Conflict Scale which has established reliability, good psychometric properties and is short (16 items) [31]. It has been used to evaluate a range of decision aids [1].

*Measures of knowledge and realistic expectations *about labour analgesia options and the benefits and risks of these options will be specific to this project. Thus we will need to develop, and test these measures as part of the project.

*Anxiety *will be measured by the state component of the short Spielberger anxiety scale which has been extensively used and validated [30,32]. We do not anticipate the decision aid will increase women's anxiety but it is important to document any changes in anxiety associated with the decision aid.

*Satisfaction *with analgesia decisions will be assessed using the Satisfaction with Decision Scale – a very brief six item scale with high reliability was developed specifically to assess satisfaction with health care decisions [33].

Satisfaction with the decision and anxiety will be measured again at 12–16 weeks postpartum. This interval was chosen to avoid the potential bias arising from questioning women still in the hospital who may feel a disloyalty to their caregivers by a critical appraisal and whose opinions have been shown to be more positive and short-lived than those obtained further out from the birth itself [34]. At that time we will also ask about exposure to the decision aid (to assess contamination), support during labour and use of pain relief methods prior to hospital admission. These issues will be further explored in the sample selected for in-depth interview.

#### Secondary outcomes

##### Service utilisation outcomes

The aim of the decision aid is to assist preference elicitation, and not to influence the direction of the decisions taken. Nevertheless, it is important to collect service utilisation and pregnancy outcome data so we will record and compare the pain relief methods used by women in all arms of the study, as well as recording and comparing rates of pregnancy complications and perinatal outcomes. The latter will be obtained (with informed consent) from the existing computerised obstetric database and include: medical or obstetric complications, induction or augmentation of labour, mode of delivery (vaginal, emergency or planned CS), enrolment to delivery interval, gestational age, birthweight, Apgar scores, perinatal deaths, Neonatal Intensive Care Unit admission and length of stay.

### 11 statistical issues

#### Sample size

The planned sample size is 600 women, with approximately 200 women to be recruited to each arm of the trial. Based on data for 2001 from the tertiary obstetric hospital where the study will be conducted, about 1500 primiparous women give birth to singleton infants after 36 weeks gestation and 92% use some form of analgesia. We anticipate that at least 50% of women will be both eligible and willing to participate.

The sample size calculations for the trial (significance 0.05, power 0.8) are based on the mean difference in the decisional conflict scale between any two arms of the trial. The effect of decision aids on this scale is documented and effect size data are available [1]. Meta-analysis of four randomised controlled trials comparing a decision aid to a pamphlet and that report a mean difference in decisional conflict gives a pooled mean difference of -4.35, 95%CI -6.8, -1.9 (on a scale ranging from 0 lowest to 100 highest decisional conflict; median standard deviation 13.0) [35-38]. Assuming a mean difference of -4.35 and standard deviation 13.0, we will need about 141 women in each arm of the trial to demonstrate a difference in decisional conflict.

Approximately 20% of primiparous women have a caesarean section (6% before labour and 14% after labour has commenced) [12]. Some of these women will lose their options for analgesia, although some may have extensive use of analgesic agents prior to caesarean section (CS). We plan to conduct an a priori sub-group analysis that excludes women who lose their options for analgesia (defined as a CS planned after randomisation, an emergency CS within 1 hour of arriving in labour or those who receive a therapeutic epidural) as these women may have different satisfaction, anxiety and decisional conflict outcomes. We will inflate the sample size estimate by 20% (from 141 to 169) to ensure sufficient power in the sub-group analyses. A further inflation of 15% for loss to follow-up, gives the final sample size of at least 195 women in each arm of the trial.

If there are no significant differences in outcome for the two decision aid groups (with or without the audio-component), the decision aid groups will be pooled giving two women with the intervention for each woman in the pamphlet group thereby increasing the power to detect differences between the decision aid and the pamphlet.

#### Data analysis

Analyses will be by intention to treat, including withdrawals and losses to follow-up firstly of all women randomised and then excluding women who lose their options for analgesia. Study groups will be compared in terms of baseline characteristics. As this is a randomised trial, we would anticipate minimal differences in baseline characteristics. If however, important differences are found, these potential confounders will be adjusted for in the analysis of outcomes. For the primary outcomes, the mean score for each measure for each group will be compared using t-tests. If adjustment for confounders is needed a multiple linear regression model will be used. The secondary outcomes will be compared using chi-squared tests of significance for categorical data and t-tests for continuous data. If adjustment for confounding is necessary logistic regression and multiple linear regression will be used respectively.

### 12 Ethical considerations

This work involves the development of a decision aid for the management of pain in labour and childbirth. Women must decide between a range of non-pharmacological and pharmacologic methods of pain relief. However this decision must be made in the context of the likely analgesic effects of each option, the risk of complications and adverse obstetric effects, and maternal preference for relief of pain. There are currently no evidenced based materials available. We therefore expect this project to be beneficial for participating women. A systematic review of decision aids found they improved knowledge without increasing anxiety. Nevertheless we will measure anxiety levels at baseline and follow-up to document any adverse effects. A trained research nurse will interview all women and obtain written informed consent. Women will be encouraged to discuss any concerns/anxiety with the research nurse and/or with their usual antenatal care provider. Women will be reassured that they are able to withdraw from the study at any time with no adverse effects on their pregnancy management. Participation will require women to complete self-report questionnaires during and after pregnancy. Working through the decision aid will take approximately 30 minutes and review of their preferences or outstanding questions will be at a routine antenatal visit. Therefore we do not consider this to be an excessive burden on their time.

The study has been approved by the Central Sydney Area Health Service Ethics Review Committee (Protocol no. X02-0247) and the University of Sydney Human Ethics Committee (Ref No. 3419). This project is funded by a nationally competitive peer-reviewed grant from the Australian National Health and Medical Research Council (No. 253635).

### 13 Confidentiality and data security

Participants in the trial will be identified by a study number only, with a master code sheet linking names with numbers being held securely and separately from the study data. To ensure that all information is secure, data records will be kept in a secure location at the University of Sydney and accessible only to research staff. As soon as all follow-up is completed the data records will be de-identified. De-identified data will be used for the statistical analysis and all publications will include only aggregated data. The electronic version of the data will be maintained on a computer protected by password. All hard copy patient identifiable data and electronic backup files will be kept in locked cabinets, which are held in a locked room accessed only by security code and limited staff. Data files will be stored for seven years after completion of the project as recommended by the NHMRC. Disposal of identifiable information will be done through the use of designated bags and/or a shredding machine.

## Competing interests

The author(s) declare that they have no competing interests.

## Authors' contributions

CR, CRG, LT and KM were involved in the conception and design of the study. CR, NN and CRG were responsible for the drafting of the protocol. All authors have read and given final approval of the final manuscript.

## Pre-publication history

The pre-publication history for this paper can be accessed here:


